# Effects of L-carnitine supplementation on oxidative stress and antioxidant enzymes activities in patients with coronary artery disease: a randomized, placebo-controlled trial

**DOI:** 10.1186/1475-2891-13-79

**Published:** 2014-08-04

**Authors:** Bor-Jen Lee, Jun-Shuo Lin, Yi-Chin Lin, Ping-Ting Lin

**Affiliations:** 1 The Intensive Care Unit, Taichung Veterans General Hospital, Taichung 40705, Taiwan; 2 School of Nutrition, Chung Shan Medical University, Taichung 40201, Taiwan; 3 Department of Nutrition, Chung Shan Medical University Hospital, Taichung 40201, Taiwan

**Keywords:** L-carnitine, Oxidative stress, Antioxidant enzymes, Coronary artery disease

## Abstract

**Background:**

Cardiovascular disease is the leading cause of death worldwide. Higher oxidative stress may contribute to the pathogenesis of coronary artery disease (CAD). The purpose of this study was to investigate the effect of L-carnitine (LC, 1000 mg/d) on the markers of oxidative stress and antioxidant enzymes activities in CAD patients.

**Methods:**

We enrolled 47 CAD patients in the study. The CAD patients were identified by cardiac catheterization as having at least 50% stenosis of one major coronary artery. The subjects were randomly assigned to the placebo (n = 24) and LC (n = 23) groups. The intervention was administered for 12 weeks. The levels of serum LC, plasma malondialdehyde (MDA), and erythrocyte antioxidant enzymes activities [catalase (CAT), superoxide dismutase (SOD), glutathione peroxidase (GPx)] were measured before and after intervention.

**Results:**

Thirty-nine subjects completed the study (placebo, n = 19; LC, n = 20). After 12 weeks of LC supplementation, the level of MDA was significantly reduced (2.0 ± 0.3 to 1.8 ± 0.3 μmol/L, *P* = 0.02) and the level of LC (33.6 ± 13.6 to 40.0 ± 12.0 μmol/L, *P* = 0.04) and antioxidant enzymes activities [CAT (12.7 ± 5.5 to 13.1 ± 5.8 U/mg of protein, *P* = 0.02), SOD (14.8 ± 2.9 to 20.7 ± 5.8 U/mg of protein, *P* < 0.01), and GPx (20.3 ± 3.4 to 23.0 ± 3.1 U/mg of protein, *P* = 0.01)] were significantly increased. The level of LC was significantly positively correlated with the antioxidant enzymes activities (CAT, *β* = 0.87, *P* = 0.02; SOD, *β* = 0.72, *P* < 0.01).

**Conclusion:**

LC supplementation at a dose of 1000 mg/d was associated with a significant reduction in oxidative stress and an increase in antioxidant enzymes activities in CAD patients. CAD patients might benefit from using LC supplements to increase their anti-oxidation capacity.

**Trial registration:**

Clinical Trials.gov Identifier: NCT01819701.

## Background

Cardiovascular disease is the leading cause of death worldwide [1] and the second most common cause of death in Taiwan. Research has demonstrated that higher levels of oxidative stress play an important role in the development of coronary artery disease (CAD) [2]. Consequently, administering antioxidants to CAD patients might improve their outcomes and prevent the recurrence of CAD [3].

L-carnitine (LC) is a non-protein amino acid (β-hydroxy-γ-trimethyl-amino-butyric acid), that is synthesized from the essential amino acids lysine and methionine [4]. LC facilitates β-oxidation of long-chain fatty acids, participates in metabolism of branched chain amino acids, and stabilizes cellular membranes [4,5]. Many *in vitro* and animal studies have reported that LC is a free radical scavenger, which protects antioxidant enzymes from oxidative damage [6-9]. In a human study, Cao et al. [10] administered LC supplement (2000 mg/d) to healthy volunteers and observed that LC significantly increased the levels of antioxidant enzymes activities, suggesting that LC might be useful for treating chronic illnesses. Additionally, some clinical trials have attempted to treat ischemic heart disease and other clinical conditions of myocardial ischemia patients with higher doses (≥2000 mg/d) of LC supplementation, and the results show that LC has a protective effect on cardiac metabolism and function after intervention [11-13]. However, little information has been published about the effect of LC on antioxidant status in CAD patients. It would be worthwhile to know whether LC should be a dietary supplement for CAD patients. Therefore, the purpose of this study was to investigate the effect of LC supplements (1000 mg/d) on the markers of oxidative stress and antioxidant enzymes activities in CAD patients.

## Methods

### Participants

This study was designed as a single blind, randomized, parallel, placebo-controlled trial. CAD patients were recruited from the cardiology clinic of Taichung Veterans General Hospital, which is a teaching hospital in central Taiwan. CAD was identified by cardiac catheterization as having at least 50% stenosis of one major coronary artery or receiving percutaneous transluminal coronary angioplasty (PTCA). The patients with diabetes, liver, or renal diseases were excluded to minimize the influence of other cardiovascular risk factors. The patients under medications therapy, such as acenocoumarol, thyroid hormone, and warfarin, and those currently receiving vitamin supplements were also excluded. Informed consent was obtained from each subject. This study was approved by the Institutional Review Board of Taichung Veterans General Hospital, Taiwan.

With a sample size calculation, we expected that the change in the levels of antioxidant enzymes activities would be 5.0 ± 7.0 U/mg of protein after LC supplementation; therefore, to achieve a desired power of 0.8 with α value of 0.05, the minimum sample size was calculated to be 18 subjects in each intervention group. We enrolled 47 CAD patients in this study and used a random numbers table to randomly assign the subjects to the placebo (n = 24) or to the LC [1000 mg/day, n = 23] groups. The LC and placebo (starch) capsules were commercially available preparations (New Health Taiwan Co., Ltd.). The intervention was administered for 12 weeks. The subjects were instructed to take two capsules daily (LC supplements 1000 mg/d, 500 mg/b.i.d). To monitor compliance, the researchers reminded subjects to check the capsules bag every 4 weeks to confirm that the bag was empty, and we measured the level of serum LC before and after intervention. The age, blood pressures, and smoking, drinking, and exercise habits of the subjects were recorded. The body weight, height, and waist circumferences were measured, and the body mass index (kg/m^2^) was calculated.

### Blood collection and biochemical measurements

Fasting venous blood samples (15 mL) were obtained to estimate the hematological and vitamin status. Blood specimens were collected in Vacutainer tubes (Becton Dickinson, Rutherford, NJ, USA) that contained EDTA as an anticoagulant or that contained no anticoagulant as required. Serum and plasma were prepared after centrifugation (3,000 rpm, 4°C, 15 minutes) and were then stored at -80°C until analysis. Hematological entities (serum creatinine, total cholesterol, triglyceride, low density lipoprotein-cholesterol, and high density lipoprotein-cholesterol) were measured by an automated biochemical analyzer (Hitachi-7180E, Tokyo, Japan). In the present study, we used MDA assay as an indicator of oxidative stress marker. MDA is generated in vivo via peroxidation of polyunsaturated fatty acids and studies show MDA can predict progression of CAD and carotid atherosclerosis at 3 years [14,15]. Plasma malondialdehyde (MDA) was determined using the TBARs (thiobarbituric acid reactive substances) method, as described by Botsoglou [16]. The serum level of LC was measured by enzyme-linked immunosorbent assay (ELISA) using commercially available kits (Cusabio, Wuhan, China) according to the instructions made available from the suppliers.

The red blood cells (RBCs) samples were washed with normal saline after removing the plasma. Then, the RBCs were diluted with 25× sodium phosphate buffer for superoxide dismutase (SOD) and glutathione peroxidase (GPx) measurements, and with 250× sodium phosphate buffer for catalase (CAT) measurement. The antioxidant enzymes activities (CAT, SOD, and GPx) were determined in the fresh samples. The methods for measuring these activities have been described previously [17-19]. The protein content of the plasma and RBCs was determined based on the biuret reaction of the BCA kit (Thermo, Rockford, IL, USA). The values of the antioxidant enzymes activities were expressed as unit/mg of protein. All of the analyses were performed in duplicate.

### Statistical analyses

The data were analyzed using SigmaPlot software (version 12.0, Systat, San Jose, CA, USA). The normal distribution of variables was tested by the Kolmogorov-Smirnov test. Differences in subjects’ demographic data and hematological measurement data between the placebo and LC groups were analyzed by Student’s t-test or the Mann–Whitney rank sum test. The paired t-test or Wilcoxon signed rank test was used to analyze the data within each group before (baseline) and after intervention (week 12). For categorical response variables, differences between the two groups were assessed by the Chi-square test or Fisher’s exact test. To examine the relationships between the levels of LC and antioxidant enzymes activities after supplementation, simple linear regressions were used. Results were considered statistically significant at *P* < 0.05. Values presented in the text are means ± standard deviations (SD).

## Results

### Study participant characteristics

**Figure 1:**
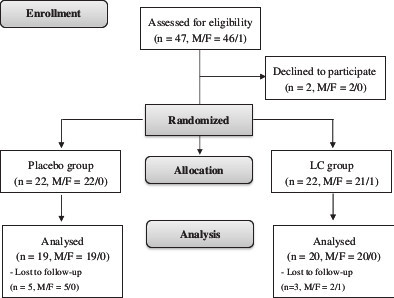
**Flow diagram.** F, female; LC, L-carnitine; M, male.

The sampling and trial profiles are summarized in Figure 1 along with the number of subjects who completed the study in each group. Table 1 shows the demographic data and health characteristics of the subjects. There were no significant differences between the two groups with respect to age, blood pressure, anthropometric measurements, hematological entities (serum creatinine and lipid profiles), and the frequency of smoking, drinking, or exercise at baseline.

**Table 1:** **Characteristics of subjects**^**1**^

	**Placebo**	**LC**	***P***
	**(n = 19)**	**(n = 20)**	**values**^**2**^
Gender (male)	19	20	-
Age (y)	72.7 ± 10.1 (74.0)	71.9 ± 10.6 (72.0)	0.80
Systolic blood pressure (mmHg)	127.3 ± 6.0 (128.0)	128.4 ± 10.4 (130.0)	0.68
Diastolic blood pressure (mmHg)	74.5 ± 4.4 (74.0)	72.3 ± 4.7 (71.0)	0.22
Waist circumference (cm)	97.7 ± 11.4 (96.0)	91.4 ± 8.4 (91.5)	0.08
Waist hip ratio	0.9 ± 0.1 (0.9)	0.9 ± 0.1 (0.9)	0.09
Body mass index (kg/m^2^)	26.0 ± 2.4 (25.6)	24.8 ± 2.6 (24.6)	0.16
Creatinine (μmol/L)	106.1 ± 44.2 (97.2)	114.9 ± 26.5 (106.1)	0.33
Total cholesterol (mmol/L)	5.0 ± 1.2 (4.6)	4.9 ± 0.7 (4.9)	0.76
Triglyceride (mmol/L)	1.7 ± 0.7 (1.6)	1.4 ± 0.7 (1.1)	0.13
Low density lipoprotein-cholesterol (mmol/L)	3.0 ± 1.3 (3.0)	3.0 ± 0.7 (3.1)	0.97
High density lipoprotein-cholesterol (mmol/L)	1.2 ± 0.2 (1.2)	1.4 ± 0.4 (1.3)	0.10
Current smoker^3^, n (%)	3 (15.8%)	4 (20.0%)	1.00
Drink alcohol^4^, n (%)	3 (15.8%)	3 (15.0%)	1.00
Exercise^5^, n (%)	17 (89.5%)	20 (100%)	0.23

^1^mean ± SD (median).

^2^values are significantly different between the placebo and LC groups.

^3^current smoker: individual currently smoking one or more cigarettes per day.

^4^drink alcohol: individual drinking one or more drinks per day regularly.

^5^exercise: individual exercising at least 3 times every week.

LC, L-carnitine.

### Effects of LC supplementation on the levels of LC, oxidative stress, and antioxidant enzymes activities

**Figure 2:**
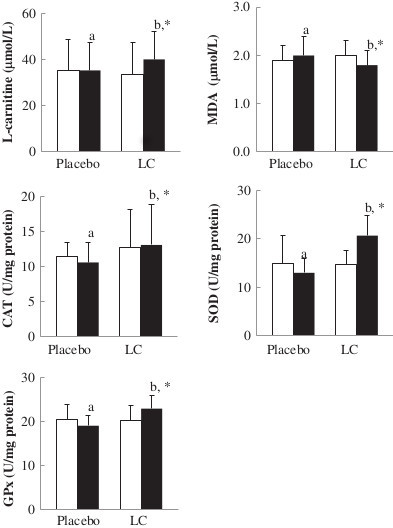
**Levels of L-carnitine, oxidative stress, and antioxidant enzymes activities after supplementation.** Data are means ± SD. □ week 0, ■ week 12. ^*^Values were significantly different after intervention within the group. ^a, b^Values with different superscripts were significantly different between the two groups. CAT, catalase; GPx, glutathione peroxidase; LC, L-carnitine; MDA, malondialdehyde; SOD, superoxide dismutase.

The levels of LC, oxidative stress, and antioxidant enzymes activities are shown in Figure 2. The subjects in the LC group had significantly lower level of MDA (1.8 ± 0.3 versus 2.0 ± 0.4 μmol/L, *P* = 0.01), higher levels of LC (40.0 ± 12.0 versus 35.2 ± 12.0 μmol/L, *P* = 0.02), and higher activities of CAT (13.1 ± 5.8 versus 10.6 ± 2.9 U/mg of protein, *P* < 0.01), SOD (20.7 ± 4.2 versus 13.1 ± 2.9 U/mg of protein, *P* < 0.01), and GPx (23.0 ± 3.1 versus 19.1 ± 2.3 U/mg of protein, *P* < 0.01) than those in the placebo group at week 12. After LC supplementation, the level of MDA was significantly reduced (2.0 ± 0.3 to 1.8 ± 0.3 μmol/L, *P* = 0.02) and levels of LC (33.6 ± 13.6 to 40.0 ± 12.0 μmol/L, *P* = 0.04) and antioxidant enzymes activities (CAT, 12.7 ± 5.5 to 13.1 ± 5.8 U/ mg of protein, *P* = 0.02; SOD, 14.8 ± 2.9 to 20.7 ± 4.2 U/ mg of protein, *P* < 0.01; and GPx, 20.3 ± 3.4 to 23.0 ± 3.1 U/mg of protein, *P* = 0.01) were significantly increased from the baseline.

The changed levels of oxidative stress and antioxidant enzymes activities after supplementation are shown in Table 2. The changed level of MDA (-0.2 ± 0.5 versus 0.1 ± 0.5 μmol/L, *P* = 0.03) was significantly lower and the antioxidant enzymes activities (CAT, 2.0 ± 6.9 versus -0.9 ± 3.4 U/mg of protein, *P* = 0.02; SOD, 5.9 ± 4.9 versus -1.9 ± 5.7 U/mg of protein, *P* < 0.01; GPx, 2.7 ± 4.4 versus -1.4 ± 3.5 U/mg of protein, *P* < 0.01) were significantly higher in the LC group than in the placebo group.

**Table 2:** **Changed levels of L-carnitine, oxidative stress marker and antioxidant enzymes activities after supplementation**^**1**^

	**Placebo**	**LC**	***P*** **values**^**2**^
	**(n = 19)**	**(n = 20)**	
LC (μmol/L)	-1.2 ± 20.0 (0.8)	7.6 ± 15.2 (7.2)	0.04
MDA (μmol/L)	0.1 ± 0.5 (0.0)	-0.2 ± 0.5 (-0.1)	0.03
CAT (U/mg protein)	-0.9 ± 3.4 (-1.0)	2.0 ± 6.9 (1.8)	0.02
SOD (U/mg protein)	-1.9 ± 5.7 (-1.2)	5.9 ± 4.9 (6.8)	< 0.01
GPx (U/mg protein)	-1.4 ± 3.5 (-1.3)	2.7 ± 4.4 (2.4)	< 0.01

^1^mean ± SD (median).

^2^values are significantly different between the placebo and LC groups.

CAT, catalase; GPx, glutathione peroxidase; LC, L-carnitine; MDA, malondialdehyde; SOD, superoxide dismutase.

### Correlations between LC and antioxidant enzymes activities after supplementation

The correlations between the levels of L-carnitine, oxidative stress, and antioxidant enzymes activities after supplementation are shown in Table 3. After 12 weeks of supplementation, the level of LC was significantly correlated with the antioxidant enzymes activities (CAT, *β* = 0.87, *P* = 0.02; SOD, *β* = 0.72, *P* < 0.01).

**Table 3:** Correlations between the levels of L-carnitine, oxidative stress, and antioxidant enzymes activities after supplementation

	**LC(μmol/L)**	
	***β***^**1**^	***P*** **values**
MDA (μmol/L)	-0.00	1.00
CAT (U/mg protein)	0.87	0.02
SOD (U/mg protein)	0.72	< 0.01
GPx (U/mg protein)	0.08	0.72

^1^regression coefficient.

CAT, Catalase activity; GPx, glutathione peroxidase; LC, L-carnitine; MDA, Malondialdehyde; SOD, superoxide dismutase.

## Discussion

To the best of our knowledge, this is the first clinical study to examine the antioxidant activity in CAD patients after oral LC supplementation (1000 mg/d). In this clinical trial, we have demonstrated that LC administered at a dose of 1000 mg/d for 12 weeks significantly reduced the oxidative stress and increased the antioxidant enzymes activities in patients with CAD. After 12 weeks of LC supplementation at a dose of 1000 mg/d can reduce the level of MDA by 7% and increase the activities of CAT by 16%, SOD by 47%, and GPx by 12%. Decreased RBCs antioxidative enzymes may accelerate the development of atherosclerosis [20]. Increased RBCs antioxidant enzymes activities can provide a protection against oxidative damage to the endothelial cells [21]. In the present study, there was a significant positive correlation between the levels of LC and antioxidant enzymes activities after supplementation. LC was found to be an effective antioxidant agent in cardiovascular disease models and prevent endothelial dysfunction through its antioxidant property [8,21,22]. As a result, it seems clear that LC has a protective effect against CAD, which could be ascribed to its antioxidant capacity.

Gülçin et al. [8] have reported that LC might be a good antioxidant. LC has an effect on free radicals (such as 1, 1-diphenyl-2-picryl-hydrazyl radical, superoxide anion radical, hydrogen peroxide) scavenging. LC might interfere with the reactive oxygen species formation and chelate the metal ferrous ions [8]. In the LC molecule, the carbonyl group can stabilize the free radicals formed on α-carbon with conjugation, and it protects plasma components against the toxic action of reactive oxygen species and reactive nitrogen species [8,9]. In addition, LC is also an essential cofactor of carnitine palmitoyltransferase 1 (CPT1), which allows fatty acid transport into mitochondria and the incorporation of long chain fatty acids into the β-oxidation cycle to obtain acetyl-CoA [23-25], and these substances enter the tricarboxylic acid (TCA) cycle to synthesize adenosine triphosphate (ATP). At this step of ATP synthesis, a large amount of oxygen is consumed, and the oxygen is reduced to water at the end of the TCA cycle. Then, oxygen concentration decreases and reactive oxygen species formation is also reduced [8,26]. We suggest that LC could be acting as a buffer for excessive acetyl groups in mitochondria, decreasing mitochondrial superoxide production during hypoxia or substrate excess, especially in the ischemic tissues.

During recent decades, much evidence has been acquired that support a clear association between oxidative stress and atherosclerotic plaque evolution [2,3,27-30].Oxidative stress might play a crucial role in cardiac and vascular abnormalities in different types of cardiovascular diseases, and antioxidant therapy might prove beneficial in combating these problems [3,27]. Administering LC at a higher dose (≥2000 mg) has shown a cardio-protective effect and reduced the death rate from CAD [31]. However, the Ministry of Health and Welfare in Taiwan recommends a daily dietary intake of no more than 2000 mg of LC. As a result, we tested a dose of 1000 mg/d in CAD patients and expected that the dose of LC (1000 mg/d) could be a dietary supplement for daily use. Based on the results of this study, we suggest that LC might be a useful dietary supplement for CAD to protect against excessive oxidative stress.

Regarding the safety of LC supplementation, Singh and Aslam [12] indicated that there are some side effects, such as mild nausea and vomiting after LC supplement at a dose of 2000 mg/d, but in three divided doses, supplementation might not cause any side effects. In the present study, we administered LC to CAD patients at a dose of 1000 mg/d in two divided doses (500 mg/b.i.d), and there were no clinically significant changes in the subjects’ vital signs, serum chemical values, or hematological values (such as blood urea nitrogen, creatinine, glutamic oxaloacetic transaminase, or glutamic pyruvate transaminase); additionally there were no serious adverse events, no complaints of myalgia or muscle weakness, no withdrawals due to adverse events, and no cardiovascular event or death report during and the end of the study. Therefore, we suggest that a dose of 1000 mg/d is safe for CAD patients.

Recent work has suggested that dietary LC might accelerate atherosclerosis via gut microbiota metabolites, complicating the role of LC supplementation in health [32]. We considered that the need for LC supplementation in CAD patients might be dependent on the status of LC in the body. Thus, LC is considered a “conditionally essential nutrient. Animals and human studies have shown that the content of LC was low in acute myocardial infarction and chronic heart failure [33,34]. A recent invited commentary in Mayo Clinic Proceedings clarifies that there is no good reason to suspect that, within the dose range used clinically, LC would promote atherosclerosis or otherwise compromise cardiovascular health. To the contrary, there is ample reason to conclude that carnitine is protective for vascular health [35]. As a result, it should be recommended that CAD patients with lower levels of LC and higher oxidative stress take LC supplements to increase their LC status and anti-oxidation capacity.

There are some limitations of the present study that should be mentioned. First, the number of participants was small, although we did recruit more subjects than expected. Second, this study was designed using daily LC supplements for 3 months only. Larger and longer intervention studies are needed to understand and establish the beneficial effects of a high dose of LC in patients with CAD. Further study is also needed to measure more mechanistic parameters and more direct indicators of oxidative damage, such as nitrotyrosine, myeloperoxidase (MPO), or oxidized low density lipoprotein (ox LDL) levels to understand the antioxidative mechanism of LC in CAD patients.

## Conclusions

In conclusion, we have demonstrated that LC supplementation at a dose of 1000 mg/d significantly reduced oxidative stress and increased antioxidant enzymes activities in CAD patients. CAD patients might benefit from using LC supplements to increase their anti-oxidation capacity.

## Competing interests

The authors declare that they have no competing interests.

## Authors’ contributions

BJL carried out the study, performed the data analyses, and drafted the manuscript. JSL and YCL carried out the study and sample analyses. PTL conceived of the study, participated in its design, and coordination, and helped to draft the manuscript. All authors read and approved the final manuscript.
